# Heavy Metal Assessment in Feathers of Eurasian Magpies (*Pica pica*): A Possible Strategy for Monitoring Environmental Contamination?

**DOI:** 10.3390/ijerph18062973

**Published:** 2021-03-14

**Authors:** Tiziano Iemmi, Alessandro Menozzi, Marcos Pérez-López, Giuseppina Basini, Francesca Grasselli, Simonetta Menotta, Paolo Serventi, Simone Bertini

**Affiliations:** 1 Department of Veterinary Science, University of Parma, Strada Del Taglio 10, 43126 Parma, Italy; tiziano.iemmi@unipr.it (T.I.); giuseppina.basini@unipr.it (G.B.); francesca.grasselli@unipr.it (F.G.); paolo.serventi@unipr.it (P.S.); simone.bertini@unipr.it (S.B.); 2 Toxicology Unit, Veterinary School, University of Extremadura, Avenida de la Universidad, 10003 Cáceres, Spain; marcospl@unex.es; 3 Istituto Zooprofilattico Sperimentale della Lombardia e dell’Emilia Romagna, via Pietro Fiorini 5, 40127 Bologna, Italy; simonetta.menotta@izsler.it

**Keywords:** bioindicator, wild bird, Ni, Pb, Cd, Hg, oxidative stress, lipid peroxidation

## Abstract

In the present study, the Eurasian magpie (*Pica pica*), was evaluated as a possible bioindicator of environmental pollution by heavy metals (HMs). Levels of Ni, Pb, Cd, and Hg in feathers of 64 magpies (31 males and 33 females) were measured by ICP-MS technique. Plasmatic biomarkers of oxidative stress (OS) were also assessed. The birds were captured in the province of Parma (Italy), in different capture sites within 1 km from urban area (UZ), and farther than 5 km from urban area (RZ). Median HM levels were 0.68 mg/kg (0.18–2.27), 2.80 mg/kg (0.41–17.7), <limit of detection (LOD) mg/kg (<LOD–0.25), 3.90 mg/kg (1.35–85.9) for Ni, Pb, Cd and Hg, respectively. No significant differences in HM levels were found according to sex, while Ni and Pb were significantly higher in adult compared to young birds (*p* = 0.047, *p* = 0.004). Conversely, Cd and Hg levels in young magpies resulted higher than those of adults (*p* = 0.001 and *p* = 0.004). No correlation was found between OS biomarkers and HM levels. No differences were found in HM levels according to capture area, except for Hg level, which resulted higher in magpies of RZ (4.05 mg/kg (1.35–12.7)) compared to UZ (2.99 mg/kg (1.54–85.9)). Further experiments are needed to establish whether magpie feathers could represent a suitable non-invasive tool for biomonitoring HMs in the environment.

## 1. Introduction

Heavy metals (HM) are those with atomic weight greater than the molecular weight of water, and while some, such as Fe, Zn, Cu, Se, Co, Mb, have a role in metabolic reactions, such as in coenzymes or functional macromolecules, and are defined microelements, indispensable for living organisms at micro concentrations, they may become harmful at high concentrations [1,2].

Some HM elements, however, such as Pb, Cd, As, or Hg, have no known biological functions, but are able to interfere with metabolic processes, causing acute toxicity as a result of exposures to high concentrations, as well as subacute and chronic toxicity, following a prolonged exposure to low concentrations; these HM are thus defined as toxic heavy metals (THMs) [3,4].

Even though THMs are naturally present in the environment at variable concentrations, the increase in the levels of environmental contamination by THMs is mostly due to anthropogenic release of polluting compounds [4]. These sources of THMs mostly include fumes and wastewater produced by industrial activities; pesticides, herbicides, and synthetic fertilizers employed in agriculture; timber processing and treatment; degradation of paints and plastic compounds, and the disposal of electronic components [5]. Moreover, THMs are persistent pollutants and are subjected to biomagnification, which means that they tend to accumulate progressively in the body of animals along the food chains [6].

The toxic action of THMs occur at all levels of the trophic chain, starting from the microbiota of the soil, through plants, up to the most complex organisms at the top of the food chain [4]. THMs are generally easily assimilated even at low concentrations, primarily through ingestion, but also by inhalation of micro-powders, and can even be absorbed through skin (especially Pb) [7]. After absorption, THMs tend to concentrate in specific target organs, thus promoting the biomagnification phenomenon, as it is well known to occur for Hg and Cd in aquatic ecosystems [8]. THMs are harmful because they may antagonize other elements with physiological functions (as it occurs with Pb and Ca), or interfere with the activity of bioactive molecules (e.g., enzymes, receptors, transcription factors) [9,10,11] or through the production of noxious free radicals and consequent molecular damage (primarily to cell membranes and nucleic acids) [12,13]. Furthermore, THMs often accumulate in liver or kidneys, and therefore cause damage to the excretory organs [7].

In consideration of the potential danger represented by exposure to THMs, the development of strategies aimed at monitoring THM levels in the environment is of the utmost importance [14]. The measurement of the levels of THM directly from the environmental abiotic matrix (water, soil, air) is not considered an accurate or suitable method, because environmental levels can vary in time (especially in the water or in the air), and thus a sample showing low levels cannot exclude the risk of dangerous exposure to THMs; by contrast, THMs in the mineral matrix may not be necessarily transferred to the trophic chain [6,15]. This has led to the development of indirect monitoring systems, such as those measuring the levels of THMs in bioindicator species, in order to evaluate the amount of these elements in the ecosystem, with the ultimate goal of protecting public health, the integrity of the ecosystem, and to have the possibility to plan and put in place effective countermeasures [6].

An ideal bioindicator species should be widespread, easily sampled and not excessively sensitive to the compound under study, since in that case even low-level exposure could lower the local population, thus hindering monitoring activity [15].

Carnivore and scavenger birds are at the top of the food chain, and are therefore particularly exposed to bioaccumulation of toxic compounds present in the ecosystem [16]. For this reason, these animals are considered as good environmental pollutant bioindicators [17], allowing to investigate the presence of contaminants along the food chain, environmental contamination levels, and the effects on animal and human health.

Furthermore, a suitable substrate to be taken from bioindicator species should be easily collected in the field, with minimal damage to animals; birds are often studied as bioindicators, and the covering feathers can be collected by operators lacking the skill needed to perform blood sampling, with no or very little discomfort for the animals [18,19].

THM levels detected in feathers may reflect the levels in blood at the time of feather formation [18,20,21,22], indicative of exposure to THMs in a relatively short period. In addition, THMs present in the environment deposit on the surface of feathers during the period between feather formation and sampling [23], or due to preening activity [19]. Indeed, the measurement of Pb in the feathers of young herons was found to be a reliable indicator of the level of exposure to metal in the time frame relative to the eruption of the feather, and was found to be correlated with the metal concentrations in kidneys and bones [24].

Several previous works have taken into consideration the measurement of THMs in feathers of wild birds to evaluate environmental pollution levels. THM levels have been assessed in feathers of Passeriformes [25,26,27,28,29,30], birds of prey [23], herons [31], and pigeons [28].

In the present work, we assessed the levels of Cd, Ni, Pb, and Hg in the feathers of a sedentary bird species, the Eurasian magpie (*Pica pica*), which is a widespread omnivore corvid, with opportunistic predatory and scavenger habits. Moreover, biomarkers of oxidative stress (OS) were also assessed in order to assess the possible correlation between THM levels and OS in this species considering the distance from urban/industrial areas. Magpies are synanthropic sedentary birds, characterized by a small home range, (between 10 and 50 km), living in urban and rural areas, both on the plains and on the hills [32]. For these reasons, and its position at the top of the food chain [33], the Eurasian magpie may be considered an ideal subject for the biomonitoring of heavy metal pollution [34].

## 2. Materials and Methods

### 2.1. Sampling Operations

**Figure 1:**
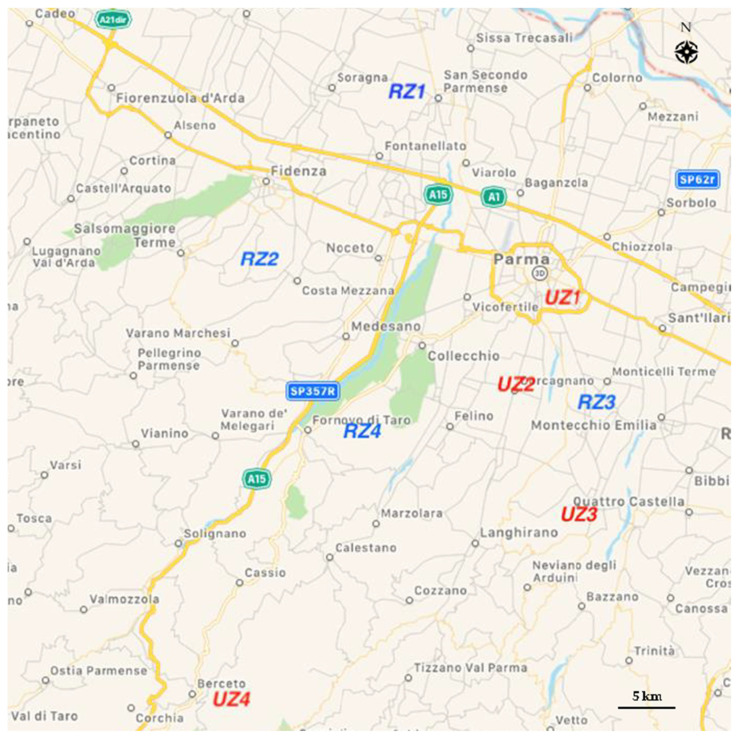
Cartographic image of the Eurasian magpie sampling zones, in the province of Parma (Italy). UZ (1–4) are sampling zones within 1 km from urban areas; RZ (1–4) are sampling zones farther than 5 km from urban areas.

Magpies (*n* = 64) were captured from January to November 2019, in 8 capture sites distributed in the province of Parma (Italy), extending from 45°02′ to 44°30′ N and from 9°57′ to 10°26′ E. The capture sites were selected with consideration of the level of urbanization, considering the distance from urban/industrial areas as an indicative factor of the presumed level of environmental pollution. Magpies captured in the 4 sites located within 1 km from urban areas and from manufacturing or industrial activities (*n* = 36), were identified as urban zone (UZ) group, while those captured in the 4 sites farther than 5 km from urban activities (*n* = 28) were identified as rural zone (RZ) group (Figure 1).

The study was approved by the Emilia Romagna Regional Ethical Committee (ISPRA Prot. 8093/T-A31 of 21 February 2019 and Region ER Det. 3751 of 1 March 2019). The authorization included trapping of target species, capture modality (with lure birds using 10 no-kill Larsen traps), and all subsequent measurements, marking and sampling procedures. Identification of species, sex, and age was performed according to a previously published technique [35], considering morphometric measurements (body weight, tarsal length, head, and culmen). All captured magpies were considered clinically healthy, and were evaluated for biometrical measurements, and marked with numbered tarsal open rings. A sample of feathers from each bird (2 external helmsman and about 10 covering feathers) was collected for the analysis of THM levels. In addition, a 1.5 mL blood sample from the right jugular vein was collected from each bird, immediately stored in heparinized tubes, and kept refrigerated at 8 °C until the delivery to the laboratory for the assessment of Hg level and biomarkers of OS.

### 2.2. Animals

The 64 magpies were 33 females (17 of the young and 16 of the adult age-class) and 31 males (12 of the young and 19 of the adult age-class).

### 2.3. THM Level Assessment

The evaluation of Cd, Ni, Pb, Hg levels was performed by means of ICP-MS technique. In order to remove external contamination by HMs from the surface of the feathers, a sequential washing process was performed prior to analytical determination, using tap water, followed by distilled water, Milli-Q water, and acetone [36,37]. A microwave-assisted acid digestion procedure adapted from previously published techniques [38,39] was carried out to obtain metal contents. Then, 0.5 g aliquots of each sample were weighed into Teflon PTFE flasks and 1.5 mL of a freshly prepared mixture of concentrated HNO_3_ (69%) and H_2_O_2_ (30%) (3:1, *v/v*) were added. The flasks were closed and left to predigest for 12 h at room temperature. The flasks were then sealed, and microwave digested (15 min with constantly increasing temperature up to 180 °C, and finally 5 min at this maximal temperature). Once the digestion was completed, the flasks were allowed to cool to room temperature, and the mixture was diluted to 10 mL with deionized water. A blank digest was carried out in the same way. All sample solutions were clear. In order to avoid losses of volatile elements, a second set of identical samples from the same individuals was dried at 80 °C in an oven till constant weight in order to calculate the percentage of humidity in each sample (average humidity of 20.85% in feathers). The accuracy of the microwave digestion method was checked by standard reference material (BCR^®^ certified reference materials—ref. 185R, Community Bureau of Reference, EU). Four replicates were done on NIST SRM 1577b bovine liver to check the accuracy, and the results were in good agreement with the certified material, with a mean recovery rate of 85–102%.

A platform collision cell inductively coupled plasma mass spectrometer ICP-MS 7900 equipped with an integrated autosampler (Agilent Tech, Santa Clara, CA, USA) was used for element detection. For an optimal nebulization of the sample, a Peltier-cooled (2 °C) cyclonic chamber (Elemental Scientific, Omaha, NE, USA) and a low-flow (0.25 mL/min) Meinhard concentric nebulizer (LGC, London, UK) were employed. Both the collision gas and the argon for the plasma have a purity of 99.999% and were supplied by Praxair (Madrid, Spain).

Each day, the ICP-MS was calibrated to obtain the highest values of intensity indicated by the ratios CeO/Ce < 2.5%, Ce^2+^/Ce < 3% and background (220) < 1 cps. The instrumental detection limits were 0.005 mg/kg for all the elements. Calibrating solutions were prepared daily from a 10 mg/L Multi-Element Calibration Standard 3 solution (PerkinElmer, Inc., Shelton, CT, USA). The same certified sample of lyophilized bovine liver previously indicated was used for quality control of the analytical procedure. The limit of detection (LOD) and of quantification (LOQ) were determined according to the ICH-Q2 guidelines on method validation (ICH 2005), after analyzing repeated blanks with the same procedure used for the samples and determining the standard deviation. The final values of both parameters were calculated taking into account the samples dilution factor and the weight and were in all cases lower than 0.003 and 0.009 mg/kg for LOD and LOQ, respectively. The coefficients of variation for replicate samples (*n* = 5) were determined to be lower than 5.3%. All samples were run in batches that included analytical blanks.

For the evaluation of Hg blood level, an aliquot of 0.7 mL of whole blood was transferred to a 50 mL Digi-Tube for subsequent digestion with Digi-Prep (SCP Science) mode. Then, 10 mL of concentrated nitric acid was slowly added to the sample tubes, allowed to stand for 15 min at room temperature, and then subjected to mineralization at 75 ± 10 °C overnight. The samples were cooled down and 20 mL of demineralized water (HPLC grade) was added to each tube: the solution was sealed and vigorously shaken for 5 min. Then 1 mL of solution was added to 10 mL of 2% aqueous nitric acid solution with 0.5% hydrochloric acid. The solution thus obtained was used to perform the measurement in ICP-MS, following the same procedure already described for the analysis from feathers. Metal concentrations were expressed as mg/kg dry weight, since dry values are considered to be more reliable and consistent compared to wet weight values [40].

### 2.4. OS Biomarkers Analysis

Plasmatic biomarkers of OS were evaluated in all the magpies, in order to establish a possible correlation between THM levels and OS in this species. For this evaluation, lipid peroxidation was assessed by measuring malondialdehyde concentration (MDA), which is considered a reliable parameter of OS-induced damage to cell membranes; in addition, determinable reactive oxygen metabolites (d-ROMs), which are considered a reliable measure of the concentration of plasmatic oxygen reactive species, and two reactive oxygen species, superoxide anion and nitric oxide (NO), were also assessed. Enzymatic antioxidant capacity was evaluated by means of superoxide dismutase (SOD) analysis, while scavenging non-enzymatic activity was determined by means of ferric reducing antioxidant power (FRAP) assay. These biomarkers were chosen because they are commonly employed to evaluate OS in most species, including birds [41,42,43,44,45,46].

#### 2.4.1. Lipid Peroxidation

Lipid peroxidation was assessed by measuring thiobarbituric acid-reactive substances, according to a previously published method [47], and expressed as nmol/mL of MDA. Briefly, 100 µL of plasma were added to 200 µL of a solution composed of 25 mL of HCl 1 N, 75 mL of distilled water (HPLC grade), 380 mg of thiobarbituric acid, and 15 g of 15% trichloroacetic acid; then, 5 µL of a 0.2% 2,6-ditert-butyl-4-methyl-phenol solution in 95% ethanol were added. The mixture was then vortexed and heated at 80 °C for 15 min. The solution was then centrifuged for 10 min at 1210× *g*, at room temperature, and the supernatant was collected and examined with a spectrophotometer at a wavelength of 532 nm (Varian Cary UV-Vis 50, Varian Technologies Italy, Milan, Italy). The calibration curve was constructed employing increasing MDA concentrations (7.8, 15.6, 31.3, 62.5, 125, 250 nmol/mL).

#### 2.4.2. d-ROMS, Superoxide Anion, and NO Levels

d-ROMs were assessed by means of a commercial test (d-ROMs Assay, Diacron s.r.l. Grosseto, Italy). Superoxide anion levels were measured by the WST-1 test (WST-1 Assay Reagent, Sigma Chemical Co Lt, St. Louis, MO, USA) according to the guidelines of the manufacturer. The assay is based on the reducing capacity of superoxide for highly water-soluble tetrazolium salt, giving rise to a soluble formazan that can be quantitatively evaluated by a colorimetric assay [48]. NO was assessed by a colorimetric technique measuring nitrite levels, based on the formation of a chromophoric compound after the reaction with the Griess reagent [49].

#### 2.4.3. Antioxidant Activity Evaluation

SOD levels were measured by means of a commercial assay kit (SOD Assay Kit, Sigma Chemical Co Lt, St. Louis, MO, USA). Scavenging non-enzymatic activity was evaluated determining the reducing ability of the plasma samples by a previously published technique called FRAP assay [50]. The FRAP assay measures the change in absorbance at 620 nm due to the formation of a blue-colored Fe^2+^-tripyridyltriazine (TPTZ) compound from colorless oxidized Fe^3+^ form by the action of electron donating antioxidants. Briefly, 20 μL of plasma were mixed with 20 μL of distilled water, and with 260 μL of FRAP reagent in each well of a 96-well plate. FRAP reagent was freshly prepared by mixing 25 mL acetate buffer (0.3 M; pH 3.6), 2.5 mL TPTZ (10 mM in 40 mM HCl), and 2.5 mL FeCl_3_·6H_2_O (20 mM). Aqueous solutions of known Fe^2+^- (FeSO_4_·7H_2_O) concentration in the 100–1000 μM range were used for the calibration curve. The absorbance was recorded with Multilabel Counter Victor3 (Perkin Elmer, Boston, MA, USA) at 620 nm after a 30-min incubation at 37 °C

### 2.5. Statistical Analysis

Statistical analysis was performed by means of GraphPad Prism ver.7 software (GraphPad Software Inc., La Jolla, CA, USA). All data were tested for normality by means of the Kolmogorov–Smirnov test, and were expressed as median and range. Mann–Whitney *U* test was employed to evaluate the significance of the difference among groups of data, according to different sampling zone, sex, and age-class. Linear regression analysis and Pearson correlation coefficient analysis were used to assess the correlation between THM and OS biomarker level observed.

## 3. Results and Discussion

### 3.1. THM Levels in Feathers

The median levels of Ni, Cd, Pb, and Hg in feathers of Eurasian magpies were, respectively: 0.68 mg/kg (0.18–2.27), <LOD mg/kg (<LOD–0.25), 2.80 mg/kg (0.41–17.7), 3.90 mg/kg (1.35–85.9).

Several previous works have already taken into consideration the measurement of HM levels in feathers of wild continental sedentary birds as a strategy to evaluate environmental pollution.

Magpie feathers were previously assessed for HM levels in a study conducted in Poland [29]. HM concentrations were considerably higher compared to our study, since Pb levels were 5–10 µg/g in samples from low-pollution areas, and 50–250 µg/g samples from highly polluted areas, while Cd ranged from 0.5 to 10 µg/g according to sampling zones.

In a more recent work, Pb and Cd levels were measured in feathers of magpies collected in Iran [46]. In this study, Pb and Cd levels were also higher than those found in our research, with mean Pb and Cd levels of 9.29 and 1.58 µg/g, respectively.

Two previous studies conducted in Malaysia in 2016 and 2017 evaluated the levels of Pb, Ni, and Cd in the organs and feathers of *Corvus splendens*, a species belonging to the same family of corvids as *Pica pica*; a significant positive correlation between the concentrations of these HMs in the feathers and in internal organs was found [25,26]. The levels of Pb and Ni detected in crow feathers in these studies were considerably higher than in our study; it should be considered, however, that these previous studies were conducted in a very densely populated area, characterized by a huge industrial development, conditions that involve a high level of HM pollution in the environment [26]. Since *P. pica* and *C. splendens*, are both opportunist omnivores which share the same ecological niche, the high difference in HM levels observed might be due to the different levels of environmental contamination, and subsequent bioaccumulation of HM along the food chains, characterizing the two areas.

In a previous study on feathers of other Passeriformes, *Cyanistes caeruleus* and *Parus major,* Cd and Ni levels close to those measured in our study but higher Pb levels, were found [27]; moreover, in selected species of birds of prey (*Accipiter nisus, Athene noctua, Tyto alba, Strix aluco*) Ni, Cd, and Pb concentrations in feather samples were not dissimilar to those we have found in *P. pica,* whereas Hg levels measured in our study were about 8 times higher compared to those detected in birds of prey [23].

In addition, a study conducted in Korea on feathers of two species of heron, *Nycticorax nycticorax* and *Ardea cinerea,* showed levels of Cd similar to our study on Eurasian magpies but lower Pb levels [31].

By contrast, Pb and Cd concentrations measured in feathers of pigeons (*Columba livia*) sampled in the highly urbanized and polluted area of Paris [28], were considerably higher than those observed in our study.

The high variability of THM levels observed in different studies, may be related to the discrepancies in the levels of environmental contamination of the study areas, but it is likely heavily influenced by the ecological niche of the different bird species. Indeed, while *C. splendens* and *P. pica* are omnivorous opportunistic birds, all the others studies involved granivorous [28], granivorous-insectivorous [27], piscivorous [31], or strictly carnivorous species [23], thus it might be difficult to compare HM levels among species with very different feeding habits, since the position of a species in the food chain is able to greatly influence HM levels by means of the biomagnification phenomenon [51].

No significant differences were found in the levels of THMs in the feathers between male and female specimens. This result is in agreement with what was previously observed in a previous study on the same species [30]. On the contrary, age seems to have an influence on THM accumulation in Eurasian magpies, since Pb and Ni levels were significantly lower in feathers of young subjects than in adults: 2.07 mg/kg (0.41–5.82), and 0.54 mg/kg (0.18–2.27) vs. 3.91 mg/kg (0.67–17.66), and 0.54 (0.18–2.27), respectively, (*p* = 0.003, and *p* = 0.047) (Table 1).

Although no differences according to age were detected in a previous study on HM levels in magpie feathers, this result is in accordance with what was observed in studies on other bird species [25,26,52]. Indeed, adult birds tend to have higher food intake due to the increased energy requirements needed for reproductive activity compared to immature subjects, and are thus potentially more exposed to food-borne contaminants [26].

By contrast, significantly higher levels of Cd (*p* = 0.0002) and Hg (*p* = 0.0044) were detected in feathers of young magpies compared to adults; Cd level was 0.16 mg/kg (0.12–0.20) in young subjects vs. <LOD mg/kg (<LOD–0.25) in adults, and Hg level was 4.58 mg/kg (1.54–85.9) vs. 3.26 mg/kg (1.35–12.7). A possible reason for this difference might be that young *P. pica* subjects are more prone to exploiting anthropogenic food sources (such as garbage and waste) due to the lower predatory skills compared to more experienced animals [35], and are therefore more exposed to contamination by some HMs, such as Hg and Cd, that might be more abundant in waste disposal sites. The use of Hg, in particular, was strongly restricted in production processes following the application of European Commission Regulation n° 1102/2008 [53], and progressively dismissed for civil use following EU Regulation n° 852/2017 [54]. The higher Hg levels in feathers of young magpies, together with the huge variability in Hg concentrations measured in this study, could therefore be related to an increased probability of exposure to Hg-containing materials like old batteries, paints, or thermometers in urban waste [55]. Indeed, one sample showed a very high Hg concentration (85.9 mg/kg), suggesting that sporadic exposure to material with high Hg content might be relevant.

No significant differences were instead found when comparing levels of Ni, Cd, and Pb in magpie feathers of UZ vs. RZ groups (Table 2). Surprisingly, the Hg level in the feathers of magpies captured in RZ was significantly higher compared to that of birds of UZ group 4.05 mg/kg (1.35–12.7) vs. 2.99 mg/kg (1.54–85.9) (*p* = 0.005).

The lack of a positive correlation between the level of urban activities of the two sampling areas and THM concentrations measured in magpie feathers raises some doubts about the real usefulness of this technique in order to evaluate environmental pollution by HMs.

This finding is in fact in contrast with a previous study, in which Pb and Cd levels in magpie feathers were significantly higher in highly industrialized capture areas compared to other unpolluted areas [29]. It is worth noting, however, that in the study conducted in Iran [30], no significant differences in Pb or Cd levels according to the level of urbanization of the sampling areas were found, in accordance to what was observed in our study.

It should, however, be taken into consideration that THM levels detected in our study are low in comparison to most previous similar studies, and this may indicate a low level of pollution by HMs in the sampling zones. It can be inferred that, with low levels of HM pollution in the environment, this biomonitoring method may be not sensitive enough to reveal small differences. Indeed, in the study conducted in Poland [29], Pb levels ranged from 5 µg/g in samples collected in unpolluted zones to 250 µg/g in samples from highly industrialized zones, so a huge difference in HM contamination of sampling areas might have been detected despite a possible low sensitivity of the technique.

### 3.2. OS Biomarkers and Hg Blood Levels

Biomarkers levels of OS are summarized in Table 3. MDA levels in females were significantly higher than in males: 23.95 mmol/L (1.10–81.60) vs. 18.40 mmol/L (1.30–53.00) (*p* = 0.036, data not shown).

A strong negative correlation was detected between superoxide anion and SOD levels (r = −0.84, *p* < 0.001). This result is not surprising, given that SOD is the enzyme that converts the highly reactive superoxide anion into hydrogen peroxide, and could thus be depleted in presence of high superoxide levels. No significant differences were found in OS biomarker levels comparing UZ and RZ groups, except for FRAP values which were higher in magpies of UZ than of RZ: 320.9 µM (106.4–1727) vs. 258.30 µM (98.9–630.2), respectively.

No correlation was demonstrated between the levels of THMs and biomarkers of OS taken into consideration. It seems, however, unlikely that such a low level of contamination by THMs could be enough to influence OS biomarkers in the studied birds.

Indeed, previous studies have investigated the presence of a correlation between THM levels and OS in birds. These studies seem to suggest that OS might not be triggered if THM are below a certain level [43,44]. Moreover, a different sensitivity to OS caused by exposure to THM, such as Pb or Hg, was found among diverse avian families [44,46]. The median Hg level in blood of magpies was 0.01 mg/kg; Hg level in blood samples was not significantly correlated with the concentration measured in feathers. This result is not surprising since the blood Hg level reflect a recent exposure to the metal, whereas the concentration in the feathers is related to the Hg accumulation during the time of the eruption phase of the examined feathers [23]. Hg blood level of adult specimens resulted significantly higher than that of young specimens: 0.02 mg/kg (<LOD–0.04) vs. 0.01 mg/kg (<LOD–0.04), *p* = 0.012.

The THM concentrations detected in our study were probably below the threshold level needed to significantly hinder the balance between oxidant and anti-oxidant factors. Accordingly, the median level of Hg in blood of magpies detected in our study was lower compared to the concentrations able to induce a relevant alteration of OS biomarkers in eagle owls (*Bubo bubo*) and griffon vultures (*Gyps fulvus*) [43,44].

## 4. Conclusions

In conclusion, the result of the present study suggests that the assessment of THM levels in the feathers of Eurasian magpies could represent an easy, non-invasive and humane method to obtain information on environmental pollution, since this species has sedentary habits, and the levels of these metals in feathers are detectable and measurable and are surely related to their presence in the environment. The lack of a correlation between the level of urbanization of the sampling zone and the THM concentrations in feathers may suggest a low sensitivity of this method in the presence of a low degree of pollution. No correlation was found between OS biomarkers and THM levels either, suggesting that a low-level exposure to these contaminants could be insufficient to cause OS in this bird species.

This study has some limitations, since the existence of a correlation of THM levels in feathers and internal organs was not assessed, and an evaluation of THM concentrations in samples of soil or food sources collected in the environment was not performed.

Further experiments are needed to better evaluate the real efficacy of this biomonitoring technique, for example, by evaluating THM levels in birds collected in more polluted areas. Additionally, it would be of great interest to investigate the existence of a correlation between THM levels in feathers of magpies and in samples of soil or in earthworms, which represent also one of the main preys of these birds.

## Figures and Tables

**Table 1:** Toxic heavy metal (THM) levels (mg/kg) in feathers of Eurasian magpies of different age-class. All data are expressed as median and range. LOD = limit of detection.

Age Class	*n*	Pb	Ni	Cd	Hg
Young	30	2.07 (0.41–5.82)	0.54 (0.18–2.27)	0.16 (0.12–0.20)	4.58 (1.54–85.9)
Adult	34	3.91 (0.67–17.7) ^a^	0.76 (0.23–2.26) ^b^	<LOD (<LOD–0.25) ^c^	3.26 (1.35–12.7) ^d^
Total	64	2.80 (0.41–17.7)	0.68 (0.18–2.27)	<LOD (<LOD–0.25)	3.09 (1.35–85.9)

^a^*p* = 0.004, ^b^ *p* = 0.047, ^c^ *p* < 0.001, ^d^ *p* = 0.004 adult vs. young.

**Table 2:** THM levels (mg/kg) in feathers of Eurasian magpies sampled in urban zone (UZ) and rural zone (RZ). All data are ex pressed as median and range. LOD = limit of detection.

Sampling Area	*n*	Pb	Ni	Cd	Hg
UZ	28	2.89 (0.41–17.7)	0.74 (0.20–1.39)	<LOD (<LOD–0.18)	2.99 (1.54–85.9)
RZ	36	2.60 (0.48–9.76)	0.66 (0.18–2.27)	<LOD (<LOD–0.25)	4.05 (1.35–12.7) *
Total	64	2.80 (0.41–17.7)	0.68 (0.18–2.27)	<LOD (<LOD–0.25)	3.09 (1.35–85.9)

* *p* = 0.005 RZ vs. UZ.

**Table 3:** Biomarkers of oxidative stress (OS) in blood of Eurasian magpies according to age-class. d-ROMs: determinable reactive oxygen species; MDA: malondialdehyde; NO: nitric oxide; WST: water soluble tetrazolium; SOD: superoxide dismutase; FRAP: ferric reducing antioxidant power. All data are expressed as median and range. LOD = limit of detection.

Age Class	*n*	d-ROMs (UCAR)	MDA (mmol/L)	NO (µmol/L)	WST (mABS)	SOD (U/mL)	FRAP (µmol/L)
Young	30	0.06 (<LOD–0.31)	22.2 (<LOD–44.7)	3.28 (1.74–11.5)	0.20 (0.08–0.47)	8.04 (2.17–21.4)	282.5 (106.4–1727)
Adult	34	0.07 (<LOD–0.18)	20.75 (1.30–82.6)	2.98 (1.48–3.67)	0.21 (0.09–0.51)	9.12 (2.43–18.0)	303.6 (98.9–632.2)
Total	64	0.06 (<LOD–0.31)	21.0 (<LOD–82.6)	3.01 (1.48–11.5)	0.21 (0.08–0.51)	8.28 (2.17–21.4)	293.1 (98.9–1727)

## Data Availability

Raw data are available on request addressed to the corresponding author.
